# CAR-NK cell for gynecological cancers: immune microenvironment remodeling and immunotherapeutic strategies

**DOI:** 10.3389/fimmu.2025.1705354

**Published:** 2025-12-04

**Authors:** Mingyao Huang, Siyang Wang, Huiyan Huang, Linze Li, Tian Wang

**Affiliations:** 1 School of Basic Medicine, Putian University, Putian, Fujian, China; 2 Key Laboratory of Translational Tumor Medicine, Putian University, Putian, Fujian, China; 3 Department of Anesthesiology, The Fourth Affiliated Hospital of China Medical University, Shenyang, China; 4 Department of Nursing, The Fourth Hospital of Xianyou, Putian, Fujian, China; 5 The Operation Room, The Fourth Affiliated Hospital of China Medical University, Shenyang, China; 6 Department of Obstetrics and Gynaecology, The Fourth Affiliated Hospital of China Medical University, Shenyang, China

**Keywords:** CAR-NK cells, gynecological cancers, tumor immune microenvironment, immunomodulation, immunotherapy

## Abstract

Gynecological malignancies, including ovarian, cervical, and endometrial cancers, represent a substantial global health burden, contributing significantly to morbidity and mortality among women. Despite advancements in therapeutic strategies, outcomes for many patients remain suboptimal due to challenges such as late-stage detection and resistance to standard treatments. The advent of chimeric antigen receptor (CAR)-engineered natural killer (NK) cells has introduced a cutting-edge immunotherapy option. This review provides an in-depth exploration of the development of CAR-NK cells, emphasizing their sources, design methodologies, and applications in managing gynecological cancers. It also examines current obstacles and outlines innovative strategies to improve the effectiveness and safety of CAR-NK cell-based therapies. Furthermore, we discuss prospective advancements, highlighting the importance of ongoing research and technological innovation to unlock the full potential of CAR-NK cells in the fight against gynecological cancers.

## 1 Introduction

Gynecological cancers, including ovarian, cervical, and endometrial cancers, are major causes of cancer-related illness and death among women worldwide (1). Together, these malignancies account for more than 1.4 million new cases and over 670,000 deaths each year, highlighting their substantial global health burden (2). Originating in the female reproductive system, these cancers present distinct challenges for early detection and effective treatment (3). Ovarian cancer, often described as a “silent killer,” typically lacks noticeable symptoms in its early stages, resulting in late-stage diagnoses and poor survival outcomes (4). While cervical cancer can largely be prevented through vaccines and regular screening, it remains a significant health issue in regions with limited resources, contributing to a high global burden of cancer-related deaths among women (5). Endometrial cancer, the most frequently diagnosed gynecological malignancy in developed nations, often shows early warning signs, but certain aggressive histological types and advanced or recurrent cases pose considerable therapeutic challenges (6).

Surgical procedures, chemotherapy, and radiotherapy remain the cornerstone treatments for gynecological cancers (7). While these methods often achieve favorable outcomes in early-stage cases, they face significant challenges in addressing advanced or recurrent cancers, which frequently develop resistance to conventional therapies. This resistance severely limits available treatment options and contributes to suboptimal patient outcomes (8). Consequently, there is an urgent need for novel approaches that can effectively overcome resistance, selectively target tumor cells, and deliver lasting therapeutic effects with minimal side effects (9).

The advent of immunotherapy has revolutionized cancer treatment, offering new hope for cancers that were previously difficult to manage (10). Among these innovative strategies, cell-based therapies involving chimeric antigen receptor (CAR)-engineered immune cells have garnered considerable interest (11). CAR-T cells, which are engineered T lymphocytes designed to recognize and destroy specific tumor antigens, have demonstrated remarkable success in treating blood cancers (12). However, the application of CAR-T cell therapy in solid tumors, including gynecological malignancies, has been hampered by several challenges. These include poor tumor infiltration, severe side effects, and immune-related complications, prompting researchers to investigate alternative immune cell platforms for CAR technology (13).

CAR-NK (chimeric antigen receptor-natural killer) cells have emerged as a potential next-generation immunotherapy, particularly for solid tumors like gynecological cancers (14). NK cells, as innate immune effectors, possess natural tumor-killing abilities and can identify and eliminate malignant cells without prior sensitization. Unlike T cells, NK cells are associated with a lower risk of severe immune-related toxicities, such as cytokine release syndrome (CRS) and graft-versus-host disease (GVHD), making them a safer candidate for CAR engineering (15). Moreover, CAR-NK cells can be generated from diverse sources, such as peripheral blood, umbilical cord blood, or induced pluripotent stem cells (iPSCs). This versatility supports the development of “off-the-shelf” therapies, which can reduce the logistical and financial burdens of personalized CAR-T cell manufacturing (16).

This review aims to provide an in-depth analysis of CAR-NK cell therapy for gynecological cancers, encompassing recent advancements in CAR-NK cell design, preclinical and clinical findings, and the benefits and limitations of this innovative approach. By examining the journey from research to clinical application, it seeks to illuminate the hurdles and prospects in leveraging CAR-NK cells to treat ovarian, cervical, and endometrial cancers. Additionally, the review will explore strategies to enhance the functionality and longevity of CAR-NK cells within the tumor microenvironment and assess the potential of combining CAR-NK cells with other immunotherapeutic agents to optimize therapeutic outcomes. This discussion aims to underscore the transformative potential of CAR-NK cells and stimulate further investigation into this promising avenue of cancer immunotherapy.

## 2 Production of CAR-NK cells

### 2.1 Origins of NK cells

**Figure 1:**
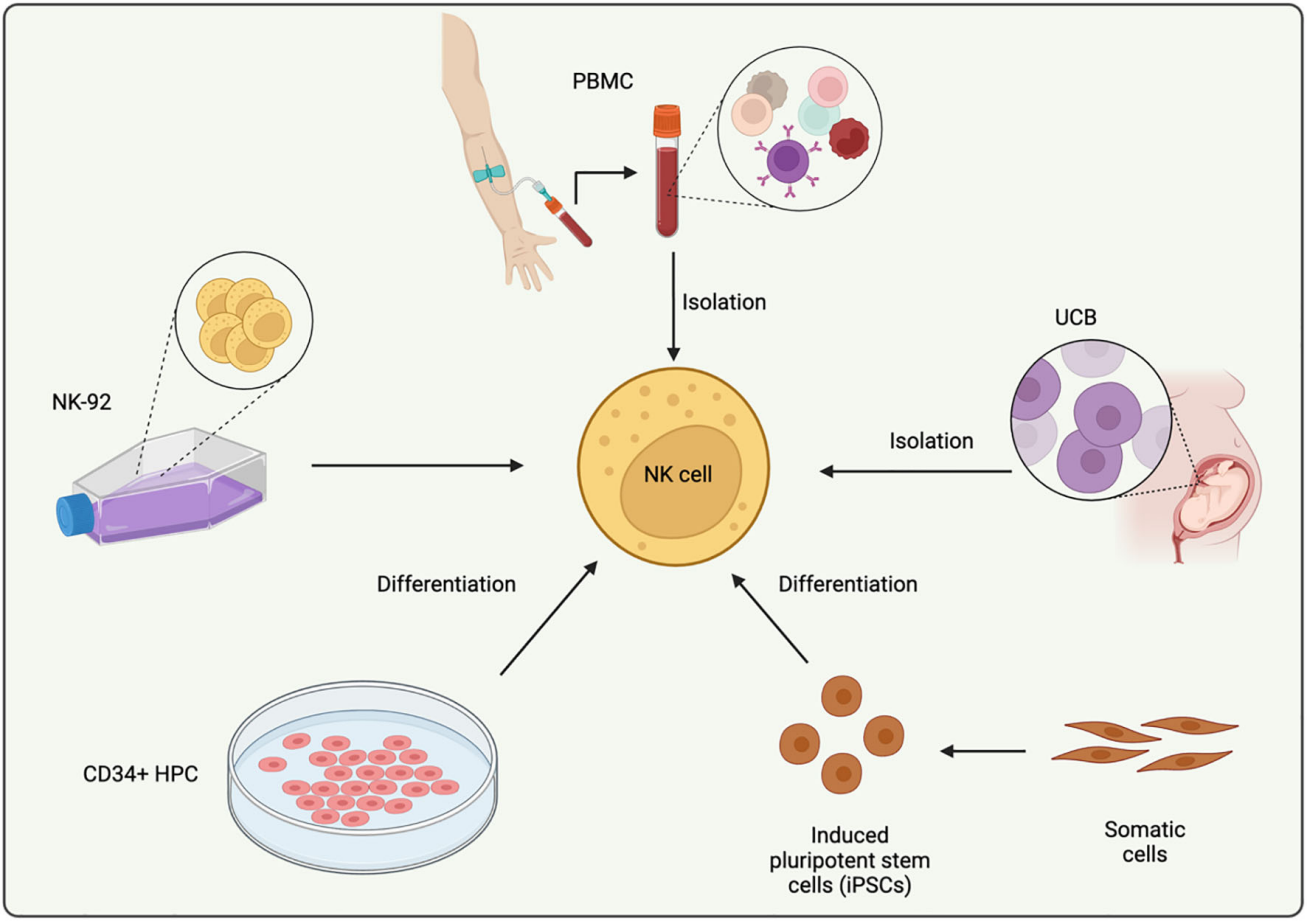
Schematic diagram of the sources of NK cells. This figure illustrates the major cellular origins used for CAR-NK production, including NK-92 cells, peripheral blood mononuclear cell–derived NK cells (PB-NK), umbilical cord blood–derived NK cells (UCB-NK), CD34^+^ hematopoietic progenitor cells (HPC-NK), and induced pluripotent stem cell–derived NK cells (iPSC-NK). Each source differs in scalability, maturity, cytotoxic potential, and suitability for “off-the-shelf” manufacturing.

CAR-NK cells offer a significant advantage over CAR-T cells by presenting a lower risk of GVHD (17–19). This characteristic makes them particularly well-suited for the development of allogeneic “off-the-shelf” therapies, which can be mass-produced, stored, and accessed for multiple patients without individual customization. Presently, CAR-NK cells that adhere to clinical-grade standards can be generated in large quantities from a range of sources, including the NK-92 cell line, peripheral blood mononuclear cells (PBMCs), umbilical cord blood (UCB), CD34+ hematopoietic progenitor cells (HPCs), and induced pluripotent stem cells (iPSCs) (**Figure 1**).

Among these sources, the NK-92 cell line is widely used in clinical trials due to its efficient proliferation *in vitro* and resistance to multiple freezing and thawing cycles (20). NK-92 cells provide an alternative to NK cells derived from patients or donors, enabling the generation of a sufficient number of cells for CAR-NK therapy without being limited by the availability of natural NK cells. These cells exhibit advantages such as high cytotoxicity, rapid division rates, the absence of inhibitory receptors, and the ease with which they can be genetically modified (21, 22). However, as a tumor-derived cell line, NK-92 cells also come with some inherent limitations, such as potential tumorigenicity, the lack of certain markers like CD16 and NKp44, and challenges in expanding the cells *in vivo* due to the irradiation required before infusion (23). Efforts to address these issues have led to modifications in NK-92 cells, such as the introduction of IL-2, resulting in variants like NK-92ci and NK-92mi (22, 24). Additionally, modifications to enhance NK-92 cell functionality have included the transduction of high-affinity CD16 and IL-2, resulting in more effective Fc receptor expression and improved cell function (22, 24).

PBMCs represent another critical source of NK cells for clinical use. NK cells can be isolated from PBMCs obtained from healthy donors using specialized kits, then activated and expanded in cytokine-rich media for preclinical research or clinical-scale production (25). CAR-NK cells generated from PBMCs often express multiple activating receptors, which allow them to expand within the patient post-infusion without the need for irradiation. PBMC-derived NK cells (PB NK cells) are typically CD56dimCD16+ cells, known for their mature phenotype, strong cytotoxic capacity, and limited proliferation ability (26). Importantly, since PB NK cells do not induce GVHD, they can be sourced from unrelated donors who are mismatched for HLA, thus offering a broader pool of potential donors and supporting the refinement of the final product (18, 27, 28). The same principles apply to NK cells derived from UCB. UCB banking allows for the selection of cells based on HLA types and NK receptor profiles. However, a key limitation is that a single UCB unit yields a relatively small number of NK cells, which can present scalability challenges. Moreover, UCB-derived NK cells tend to be less mature and have reduced cytotoxic activity compared to PB NK cells, with lower expression of key markers like CD16, KIRs, perforin, and granzyme B, while exhibiting higher levels of inhibitory receptors such as NKG2A (29). This variability in NK cell maturity and functionality presents a challenge in standardizing CAR-NK products derived from PBMCs and UCB for clinical use (30).

Beyond mature NK cells from PBMCs and UCB, CD34^+^ HPCs and iPSCs provide two additional and highly valuable sources that further expand NK cell derivation strategies. CD34^+^ HPCs, isolated from bone marrow, umbilical cord blood, or mobilized peripheral blood, can naturally differentiate into NK cells under cytokine-driven culture conditions. HPC-derived NK cells generally display strong proliferative capacity, robust responsiveness to IL-15, and potent cytotoxic activity after maturation, making them suitable for consistent and scalable production of CAR-NK cell products (31–34).

In parallel, iPSCs offer an essentially unlimited and genetically tractable source for NK cell generation. Through stepwise differentiation protocols that mimic embryonic hematopoiesis, iPSCs can be efficiently directed into NK progenitors and subsequently matured into functional NK cells with defined phenotypes. Importantly, gene engineering can be performed at the pluripotent stage, enabling the generation of homogeneous, clonal CAR-NK products with high reproducibility and batch-to-batch consistency. Multiple studies have demonstrated that iPSC-derived CAR-NK cells exhibit potent cytotoxicity, improved persistence, and excellent scalability, highlighting their promise as an ideal platform for universal “off-the-shelf” immunotherapy (31, 35, 36).

Together, these emerging sources complement NK-92, PBMC-, and UCB-derived NK cells and help build a more versatile, scalable, and standardized platform for CAR-NK cell manufacturing.

### 2.2 CAR engineering for NK cells

CAR technology was initially developed to enhance the ability of T cells to target and attack cancer cells (37). CARs are made up of three key components: an extracellular domain, a transmembrane region, and an intracellular signaling domain (38, 39). The extracellular part, typically derived from a single-chain variable fragment (scFv) of an antibody, allows for the recognition of specific antigens. Recently, smaller, high-affinity single-variable domains derived from heavy chains (VHH) have been explored as alternatives for antigen recognition (40, 41). The structure of CAR includes the scFv or VHH for antigen binding, along with a hinge region that links to the transmembrane domain, anchoring the CAR to the cell membrane (42).

**Figure 2:**
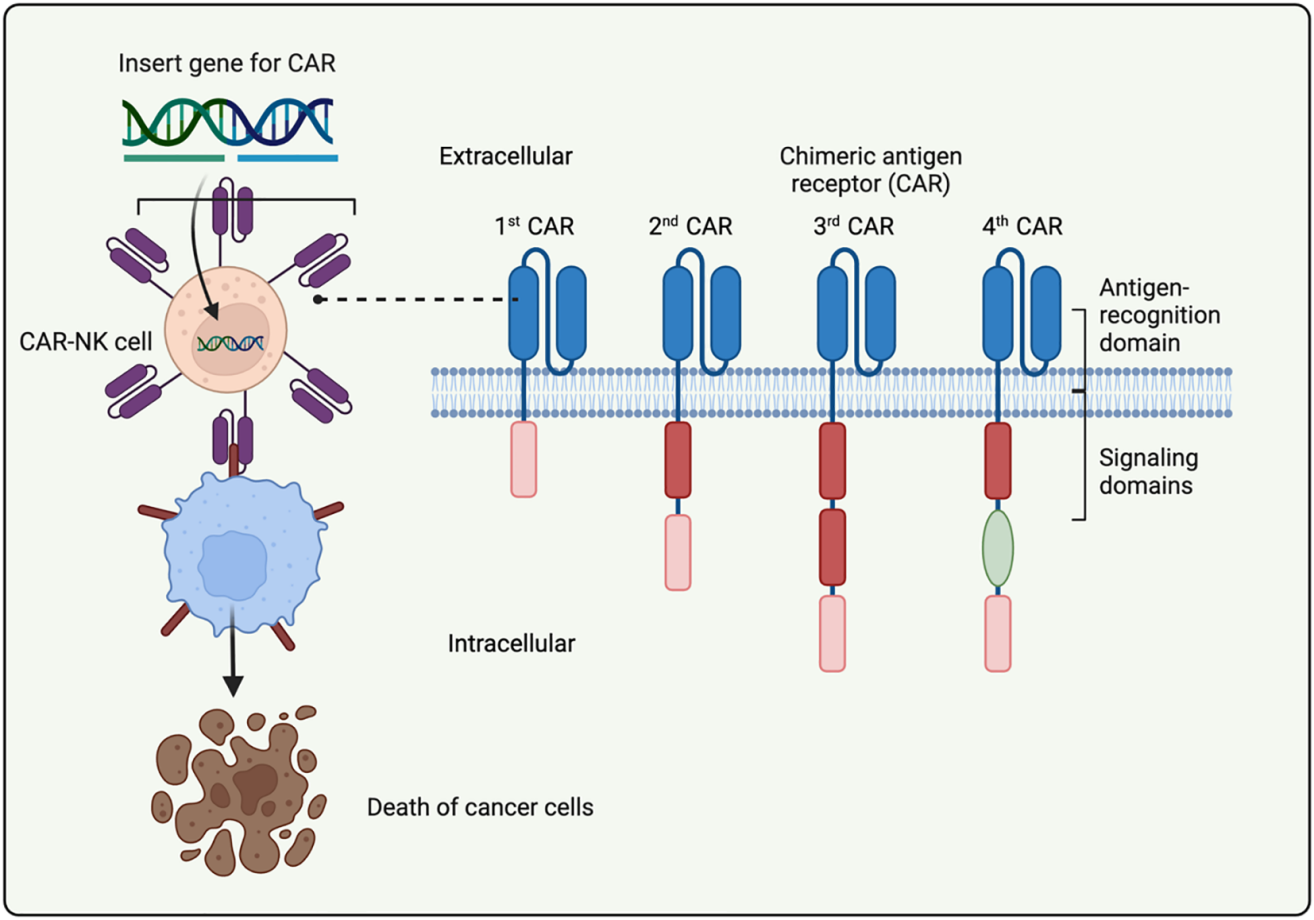
Overview of the constructs of CAR-NK cells. This schematic summarizes the architecture of CARs, including antigen-recognition domains (scFv or VHH), hinge region, transmembrane domain, and intracellular signaling motifs (e.g., CD3ζ, CD28, 4-1BB, DAP10, DAP12). The figure also highlights the evolutionary progression from first-generation CARs (CD3ζ only) to fourth-generation CARs incorporating additional costimulatory elements, cytokine expression cassettes, or safety switches for enhanced function and safety.

The intracellular domain of the CAR contains signaling elements sourced from T cell or activation receptors, which are crucial for activating engineered effector cells upon encountering the antigen (43, 44). CARs are categorized into different generations based on their design evolution. First-generation CARs only contain the CD3ζ signaling domain, but they often require additional signals for a full cytotoxic response (45, 46). Second-generation CARs add a costimulatory domain, such as CD28, CD137 (4-1BB), or CD134 (OX40), which, together with CD3ζ, enhances activation. In third-generation CARs, two costimulatory domains are included to further improve cell persistence and activation, although these have not shown clear advantages over the second-generation CARs (47). Fourth-generation CARs utilize synthetic biology approaches to improve both function and safety (48–50), incorporating engineered cytokine genes for self-activation and inducible caspase-9 systems to act as safety switches to control cell toxicity (51) (**Figure 2**).

In recent years, NK cells have emerged as a promising alternative for CAR-based therapies (52). Since NK and T cells share common signaling components like CD3ζ, CD28, and 4-1BB, CAR designs developed for T cells can be adapted for use in NK cells with proven success (53). Research using NK-specific signaling domains such as DNAX-activation protein (DAP)10 or DAP12, instead of CD3ζ, has demonstrated enhanced cytotoxicity in PBMC-derived NK cells (54–57). In a study by Li et al. (31), CAR constructs were compared in NK-92 and iPSC-derived NK cells, revealing that incorporating NK-specific transmembrane and costimulatory domains significantly improved both cytotoxicity and activation. These findings underscore the potential of refining CAR-NK designs to better suit NK cell biology and enhance anti-tumor efficacy. Overall, compared with CAR-T cells, CAR-NK cells offer several intrinsic advantages that further support their rapid development. First, NK cells can eliminate malignant cells through both CAR-dependent and natural cytotoxic pathways, enabling them to target tumor cells even when antigen expression is heterogeneous or downregulated, a limitation frequently observed in CAR-T cell therapy (25, 58). Second, CAR-NK cells exhibit a markedly lower risk of CRS and virtually no GVHD, making them substantially safer than CAR-T cells in clinical settings (59, 60). Finally, the availability of diverse NK cell sources—including PBMCs, UCB, NK-92, CD34^+^ progenitors, and iPSCs—offers greater flexibility and scalability for standardized “off-the-shelf” manufacturing compared with autologous CAR-T production, which is time-consuming and costly (61, 62).

Despite these advances, CAR-based therapies still face significant challenges, particularly antigen escape, which is a major cause of resistance to treatment (58). Bispecific CARs, which target multiple antigens or include dual-recognition domains, are being developed to enhance tumor recognition and reduce the risk of antigen evasion (63). Trogocytosis, a process in which target cell molecules are transferred to effector cells, increases the likelihood of fratricide and dysfunction in CAR-T cells (64). To mitigate this, inhibitory CARs (iCARs) targeting NK cell-specific inhibitory receptors have been incorporated into CAR-NK cells. These iCARs deliver a “do not kill” signal, allowing NK cells to avoid attacking each other. Combining an activating CAR (aCAR) targeting tumors with an iCAR for self-recognition has been shown to reduce fratricide caused by trogocytosis, thereby enhancing the function and durability of CAR-NK cells (65).

## 3 Application of CAR-NK cells in gynecological cancer treatment

**Figure 3:**
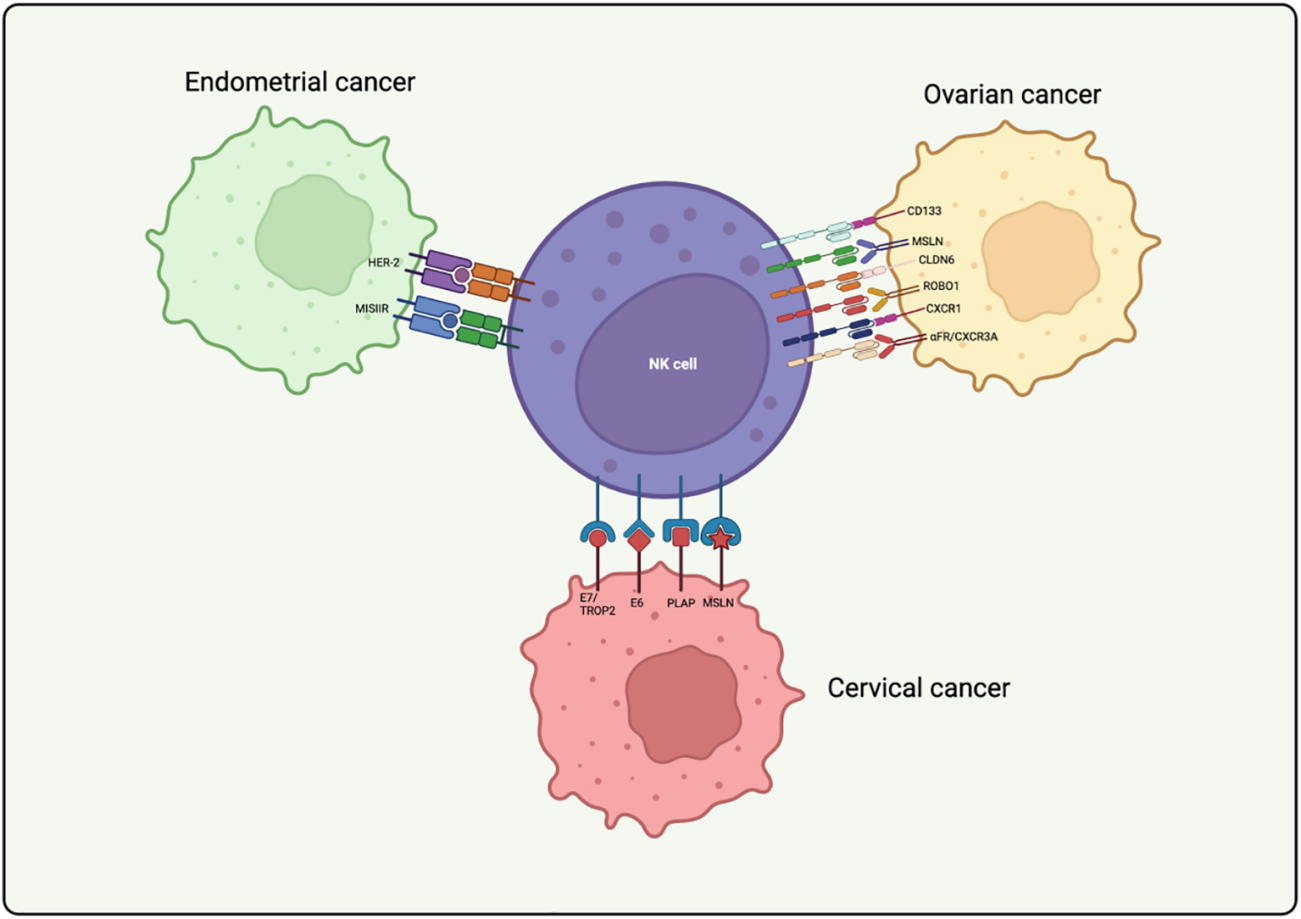
Application of CAR-NK cells in gynecological cancer treatment. The figure illustrates the targets, cancer types, and NK cell sources utilized in CAR-NK cell therapies for gynecological cancers, including ovarian, cervical, and endometrial cancers. Specific antigens such as CD133, MSLN, CLDN6, and others are highlighted, demonstrating the versatility and potential of CAR-NK cells in addressing tumor heterogeneity and improving therapeutic outcomes.

This chapter will explore the application of CAR-NK cells in different gynecological cancers (**Tables 1**, **2**) (**Figure 3**).

**Table 1:** Validated CAR-NK targets already developed in gynecological cancers.

Target	Cancer type	NK cell source	Mechanism of action	Key cytotoxicity results	Survival benefit (*in vivo*)	Ref
CD133	Ovarian cancer	NK-92	Targets CD133^+^ CSC-like populations	Selective killing of CD133^+^ tumor cells; retains activity with cisplatin	Not reported	(68)
MSLN	Ovarian cancer	NK-92	Binds membrane-bound MSLN	Strong lysis of MSLN^+^ cells	Significant prolongation of mouse survival	(73)
MSLN	Ovarian cancer	hESC-derived NK cells	Binds membrane-bound MSLN	Robust cytotoxicity *in vitro*	Suppresses tumor growth; improved survival	(74)
CLDN6	Ovarian cancer	NK-92	Recognizes CLDN6 on tumor cells	Selective killing of CLDN6^+^ cancer cells	Effective elimination of IP tumors	(80)
ROBO1	Ovarian cancer	PB-NK	Targets SLIT-ROBO axis related to metastasis	Enhanced cytotoxicity vs. unmodified NK	Not reported	(85)
CXCR1-CAR	Ovarian cancer	PB-NK	CXCR1 improves homing toward IL-8	Increased migration + cytotoxicity to IL-8^+^ cells	Not reported	(91)
αFR/CXCR3A	Ovarian cancer	NK-92	CAR-mediated killing + CXCL10-induced infiltration	Strong killing of αFR^+^ cells	Improved infiltration → better tumor clearance	(92)
E7/TROP2	Cervical cancer	NK-92	Combines HPV-E7 and TROP2 recognition	Higher cytotoxicity vs. E7-alone CAR	Not reported	(94)

**Table 2:** Potential CAR-NK targets for gynecological cancers and related CAR-T/Other CAR studies.

Targets	Cancer	CAR-T or Other CAR Developed?	Notes	Ref
E6	Cervical cancer	Yes – CAR-T using TCR-mimic nanobody	Strong antitumor activity; suitable for NK conversion	(95)
PLAP	Cervical cancer	Yes – PLAP-CAR-T	Tumor-restricted expression	(98)
MSLN	Cervical cancer	Yes – CAR-T preclinical & early clinical	Strong evidence supports CAR-NK development	(99)
HER-2	Endometrial cancer	Yes – HER2 CAR-macrophages; HER2 CAR-T widely used	Overexpressed in aggressive EC	(102)
MISIIR	Endometrial cancer	Yes – MISIIR-CAR-T	Strong tumor specificity; safe in normal tissues	(106)
ALPP	Endometrial cancer	Yes – ALPP-CAR-T clinical trial	Good safety; potential for CAR-NK	(102)

### 3.1 Ovarian cancer

CD133 (Prominin-1) is a transmembrane glycoprotein, initially identified as a marker of hematopoietic stem cells, and now recognized as a key surface marker for cancer stem cells (CSCs) in various cancers, including ovarian cancer (66). It is associated with tumor initiation, progression, chemoresistance, and recurrence (66, 67). Notably, CAR-NK cells targeting CD133 have shown selective cytotoxicity against CD133-positive ovarian cancer cells and primary tumor cells derived from ascites collections (68). Moreover, CAR-NK cells retain their cytotoxicity under cisplatin treatment, and sequential administration after cisplatin results in enhanced tumor cell killing. These findings highlight the potential of using CD133-targeted CAR-NK cells to selectively eliminate ovarian CSCs (68).

Mesothelin (MSLN), overexpressed in ovarian cancer, is another promising target for immunotherapy (69). CAR-T cells targeting MSLN have shown potent antitumor effects against ovarian cancer (70–72). Building upon this approach, CAR constructs have been adapted for NK cells, leading to the development of MSLN-targeting CAR-NK cells. For instance, Cao et al. engineered MSLN-targeting CAR-NK cells by transducing NK-92 cells with a lentiviral vector encoding an MSLN antibody. These engineered MSLN-CAR-NK cells demonstrated strong efficacy in eliminating ovarian cancer cells in both subcutaneous and intraperitoneal tumor models, significantly extending the survival of mice with intraperitoneal tumors (73). Moreover, human embryonic stem cell (hESC)-derived MSLN-CAR-NK cells have shown success in suppressing ovarian cancer progression in animal studies, with the added advantage of enabling large-scale production and cryopreservation, making the approach more cost-efficient for clinical use (74). Despite these promising results, most anti-MSLN antibodies bind to the soluble form of MSLN, which prevents effective targeting of MSLN on tumor cell membranes. To overcome this, a novel anti-MSLN antibody, 15B6, has been developed. This antibody specifically binds to the membrane-proximal region of MSLN, avoiding interaction with soluble MSLN, thus improving the antitumor activity of CAR-T cells targeting MSLN (75). The unique binding property of the 15B6 antibody enables MSLN-CAR-T cells to exhibit significantly enhanced antitumor activity compared to CAR-T cells that target shed MSLN. Such a CAR construct holds promise as a strategy to boost the antitumor efficacy of MSLN-CAR-NK cells. By avoiding interaction with shed MSLN and specifically targeting the membrane-proximal region of the MSLN protein, 15B6-based MSLN-CAR-NK cells could achieve superior tumor targeting and therapeutic potential.

In recent years, Claudin-6 (CLDN6), a membrane-associated protein, has gained attention as a potential target for cancer immunotherapy due to its high expression in several solid tumors, including ovarian, testicular, and endometrial cancers (76). CLDN6 plays a crucial role in regulating key cellular processes such as proliferation and apoptosis, with its function differing based on the tumor type, thus acting as either a tumor promoter or suppressor (77). While absent in most normal adult tissues, CLDN6 is found on the surface of a variety of cancer cells, making it an attractive therapeutic target (78, 79). Recent studies have shown that second-generation CAR-T cells targeting CLDN6, enhanced by RNA vaccines, exhibit promising preclinical efficacy in solid tumors. Additionally, the development of third-generation CLDN6-specific CAR-NK cells has shown their ability to selectively target and kill CLDN6-positive ovarian cancer cells *in vitro*, while leaving CLDN6-negative cells unharmed. *In vivo* studies further demonstrated that these CAR-NK cells effectively eliminated ovarian cancer cells in both subcutaneous and intraperitoneal models, underscoring their potent antitumor activity (80). More notably, combining CLDN6-targeted CAR-NK cells with immune checkpoint inhibitors such as anti-PD-L1 has been shown to enhance their antitumor effects, suggesting a promising synergistic approach. These advancements position CLDN6-CAR-NK cells as a potential therapeutic option for ovarian cancer, offering hope for improved treatment outcomes.

ROBO1, a transmembrane receptor for SLIT proteins, has been found to be upregulated in ovarian cancer cells (81). The SLIT-ROBO signaling pathway is crucial in promoting tumor angiogenesis and metastasis (82–84). To minimize the adverse effects associated with chemotherapy and reduce the risks inherent to allogeneic therapies, researchers have engineered ROBO1-targeted CAR-NK cells using patient-derived peripheral blood mononuclear cells (PBMCs). These CAR-NK cells targeting ROBO1 have shown greater effectiveness in eliminating primary ovarian cancer cells and releasing ovarian tumor organelles when compared to unmodified NK cells (85). Importantly, CAR-NK cells generated from autologous PBMCs offer a promising personalized approach for ovarian cancer treatment. However, challenges arise due to the variability in PBMC composition across different patients. The proportion of NK cells in PBMCs can vary significantly, ranging from 5% to 20%, with differences in their proliferative capabilities (86, 87). This variability makes it difficult to standardize the criteria for selecting NK cells that are appropriate for CAR transduction. Moreover, there is currently no consensus on the most efficient method for producing CAR-NK cells from PBMCs, as multiple techniques have been used across various studies to isolate and stimulate NK cells (88, 89).

A key challenge in CAR immunotherapy for solid tumors is the limited infiltration of CAR-modified immune cells into the tumor. Fourth-generation CAR-NK cells overcome this barrier by being genetically engineered to secrete cytokines that enhance their ability to penetrate the tumor microenvironment (90). In ovarian cancer research, CXCR1-modified CAR-NK cells were created by electroporating NK cells with mRNA constructs encoding CXCR1. This modification significantly boosted their migration toward IL-8-secreting ovarian cancer cells *in vitro*, improving tumor targeting (91). Additionally, a study developed CAR-NK cells targeting folate receptor α (αFR) and co-expressing CXCR3A. These αFR-CXCR3A-CAR-NK cells could bind to the chemokine CXCL10, increasing their infiltration into ovarian cancer cells. The study also showed that αFR-CAR-NK cells secreted IFN-γ and TNF-α, stimulating high CXCL10 expression in ovarian cancer cells, which created a positive feedback loop further promoting CAR-NK cell infiltration (92). These findings suggest that incorporating chemokine receptor engineering and cytokine secretion into CAR-NK cell designs can greatly enhance their therapeutic efficacy in solid tumors. However, translating these findings into clinical practice requires addressing safety concerns, such as off-target effects and the potential for excessive cytokine release. Additionally, optimizing the balance between chemokine receptor expression and tumor specificity is crucial to ensure targeted tumor infiltration while sparing healthy tissues. These advancements lay the foundation for more personalized and effective CAR-NK cell therapies tailored to the specific chemokine profiles of different tumors.

### 3.2 Cervical cancer

Cervical cancer is primarily caused by persistent infection with high-risk human papillomavirus (HPV), making it a prime candidate for immunotherapeutic interventions. One promising approach involves CAR-NK cells engineered to target HPV E6 and E7 antigens, which have shown potential in preclinical models. Specifically, CAR-NK cells targeting the HPV E7 antigen effectively eliminate HPV-positive cervical cancer cells, resulting in significant tumor regression. Notably, CAR-NK cells co-expressing E7 along with trophoblast cell surface antigen 2 (TROP2), a member of the tumor-associated calcium signal transducer (TACSTD) family, demonstrated considerably enhanced antigen-specific activation and cytotoxicity against cervical cancer cells compared to those expressing E7 alone (93, 94). This strategy offers a potential avenue to improve the effectiveness of adoptive cell immunotherapies for cervical cancer, an area under active investigation. Additionally, CAR-T cells incorporating T-cell receptor-mimicking nanobodies targeting HPV16 E6 have also exhibited significant antitumor effects, further supporting the feasibility of targeting E6 in cervical cancer therapy (95). Further research is needed to optimize the expression of these nanobodies in NK cells using techniques such as viral vectors or mRNA delivery to maximize CAR-NK cell activation and function.

Another promising tumor-associated antigen (TAA) in cervical cancer is placental alkaline phosphatase (PLAP), which is also expressed in ovarian, colorectal, and prostate cancers, but is minimally present in normal tissues (96, 97). Yekehfallah et al. constructed second-generation CAR-T cells with fully human scFv targeting PLAP, which demonstrated strong cytotoxicity against cervical cancer cells (98). In addition, MSLN-targeted CAR-T cells have shown potent antitumor activity against cervical cancer both *in vitro* and *in vivo*, further supporting the therapeutic potential of targeting PLAP and MSLN for cervical cancer treatment (99). These findings suggest that PLAP and MSLN may be promising targets for developing CAR-NK cells specifically aimed at cervical cancer. Future research should focus on evaluating the ability of these CAR-NK cells to infiltrate the tumor microenvironment, overcome immune suppression, and maintain their activity *in vivo*. Combining CAR-NK cell therapy with immune checkpoint inhibitors, cytokines, or other immunotherapies could further enhance their therapeutic potential, paving the way for effective treatment strategies for cervical cancer.

### 3.3 Endometrial cancer

Human epidermal growth factor receptor 2 (HER-2) is frequently overexpressed in endometrial cancer (100), and its overexpression is linked to a poor prognosis (101). A clinical trial investigated the efficacy of HER-2-targeted CAR-macrophages in treating HER-2-positive solid tumors. The results demonstrated good safety and tolerability, with disease stabilization observed in patients (NCT04660929) (102). These findings highlight the potential of HER-2-targeted CAR therapies in improving immune cell specificity and enhancing cytotoxicity against HER-2-positive tumor cells. Consequently, the development of HER-2-targeted CAR-NK cells emerges as a promising approach for treating endometrial cancer.

Furthermore, the Müllerian inhibiting substance type 2 receptor (MISIIR), a member of the transforming growth factor β (TGF-β) receptor family, is overexpressed in ovarian and endometrial cancers, while its expression is minimal in normal tissues (103, 104). This selective expression, combined with the fact that MISIIR activation induces apoptotic signals in cancer cells, makes it an attractive therapeutic target (105). Recent research has focused on the creation of a CAR targeting MISIIR. T cells engineered with a MISIIR-specific CAR showed strong antigen-specific reactivity *in vitro*, effectively recognizing and eliminating MISIIR-overexpressing endometrial cancer cells. Importantly, these CAR-T cells did not exhibit cytotoxicity against a variety of normal human cells, indicating the approach’s specificity and safety (106). These promising results open the door for exploring MISIIR as a target in CAR-NK cell therapies. Expanding this strategy to CAR-NK cells could offer a safer and more potent alternative for treating cancers, including ovarian and endometrial cancers, which overexpress MISIIR. Future clinical trials will be crucial in assessing the safety and efficacy of CAR-NK cell therapy for endometrial cancer, particularly in overcoming resistance to treatment and improving the effectiveness of current therapies.

The first clinical trial investigating anti-ALPP CAR-T cell therapy for ovarian and endometrial cancers (NCT04627740) is a single-center, open-label study targeting alkaline phosphatase placental (ALPP)-positive tumors. Preliminary results have shown that ALPP-targeted CAR-T cells provide therapeutic benefits for patients with ALPP-expressing malignancies (102). These findings suggest that CAR-modified NK cells targeting ALPP could also offer a viable therapeutic option for tumors that overexpress ALPP, such as ovarian and endometrial cancers. By leveraging the innate advantages of NK cells, including their ability to target tumor cells without prior sensitization, CAR-NK cell therapy could enhance antitumor activity and address some of the limitations observed with CAR-T cell therapies.

## 4 Strategies to overcome limitations of CAR-NK cell therapy

The first large-scale clinical trial of CAR-NK cells (NCT03056339) demonstrated their safety and significant clinical efficacy in patients with CD19+ chronic lymphocytic leukemia and B-cell lymphoma (59). As highlighted, CAR-NK cells offer distinct advantages over CAR-T cells in cancer immunotherapy. However, they face similar challenges, including antigen loss, tumor heterogeneity, and the immunosuppressive TME. Therefore, it is crucial to investigate strategies to enhance the effectiveness of CAR-NK cell therapies in the future.

### 4.1 Targeting antigens for CAR-NK cell development

CAR-NK cells have the unique ability to kill cancer cells through both CAR-dependent pathways and their inherent cytotoxic mechanisms. This characteristic allows for the engineering of CARs with mild activation signals, such as those without a costimulatory domain, enabling CAR-NK cells to predominantly utilize their natural cytotoxic abilities while minimizing CAR-induced cytotoxicity (107). Alternatively, NK cells can be engineered with a non-activating CAR, which does not directly induce cytotoxicity but enhances targeting, adhesion, and migration toward cancer cells (108). This approach capitalizes on NK cells’ natural cytotoxic functions, reducing the risk of damaging normal cells expressing the target antigen. Consequently, it expands the range of potential antigens for targeting, including HER2, EGFR, and mesothelin.

Additionally, selecting tumor-specific antigens that are highly expressed on gynecological cancer cells while showing minimal or no expression in healthy tissues is crucial. The ideal target should be abundant on cancer cells with limited presence on normal tissues, thereby minimizing off-target effects and toxicity (109, 110). Furthermore, targeting antigens involved in tumor growth or survival strengthens the therapeutic rationale (111). Given the tumor heterogeneity in gynecological cancers, identifying multiple antigens or developing multi-target CAR-NK cells may enhance efficacy and prevent tumor escape due to the downregulation of a single target (112). This strategy could improve treatment effectiveness and reduce the impact of antigen loss during tumor progression.

### 4.2 Addressing the tumor immunosuppressive microenvironment

The TME presents a significant challenge to the efficacy of immune cell-based therapies. Among the key players in the TME, cancer-associated fibroblasts (CAFs) are known to foster tumor progression by supporting various aspects of tumor growth (113). As a result, targeting CAFs could be a strategic approach to counteract the immunosuppressive effects of the TME. In a novel approach, Sakemura et al. engineered BCMA/CAF-CAR-T cells that simultaneously targeted both BCMA and CAFs. This dual-targeting strategy enhanced CAR-T cell activity and improved anti-tumor efficacy in models of human multiple myeloma (114). Similarly, optimizing the culture conditions for CAR-NK cells has shown potential in overcoming TME-induced suppression, as highlighted by previous studies on CAR-T cells (115).

The TME is rich in immunosuppressive elements, including factors like hypoxia, TGF-β, PGE-2, and metabolites such as lactate and adenosine, all of which contribute to immune cell dysfunction (116). One promising approach to counteracting these inhibitory signals involves genetically modifying NK cells to withstand these suppressive influences. For example, a reduction in TGF-β receptor II expression on NK cells has been found to alleviate TME-induced inhibition while preserving their anti-tumor activity (117). Other strategies might involve incorporating CAR designs with negative TGF-β receptors or pairing them with TGF-β blockers to enhance their effectiveness (118).

Additionally, tumor cells can evade immune surveillance by increasing the expression of ligands for inhibitory immune receptors, exploiting the immune system’s negative feedback mechanisms. Overcoming this immune evasion can be achieved through various strategies, such as knocking down receptors like NKG2A on NK cells or combining CAR therapy with checkpoint inhibitors to bolster the immune response against the tumor (119, 120). Designing CARs that target inhibitory checkpoints also shows potential (121), as shown in early studies on solid tumors (122).

### 4.3 Enhancing the persistence of CAR-NK cells

NK cells are characterized by a relatively short lifespan and a rapid turnover rate *in vivo*, which distinguishes them from long-lasting lymphocytes like T cells (123). As a result, CAR-NK cells typically have a survival span of only 1–2 weeks, limiting their effectiveness unless supported by cytokines. However, the administration of exogenous cytokines can introduce side effects, such as the activation of regulatory T cells, which might diminish the functionality of NK cells (124). Thus, it is critical to develop strategies that not only prolong the lifespan of CAR-NK cells but also enhance their performance in the TME.

Advancements in genetic engineering have made it possible to generate NK cells that express CARs and carry additional therapeutic agents, including cytokines, antibodies, and proteases, to improve their growth, migration, and ability to infiltrate tumors. For instance, IL-15 is essential for the development, survival, and activation of NK cells. CAR-NK cells engineered to express IL-15 have demonstrated increased persistence and improved anti-tumor responses *in vivo* (125). Moreover, recent studies have introduced a short hairpin RNA targeting PD-1 into BCMA-CAR NK cells integrated with an OX-40 costimulatory domain. This modification resulted in reduced T cell exhaustion and a higher proportion of memory T cells *in vitro*, compared to conventional BCMA-CAR-T cells (126). This “armored” CAR-NK cell approach shows promise for enhancing therapeutic efficacy.

Furthermore, innovative strategies also include targeting apoptotic pathways in tumor cells or employing gene-editing tools to remove pro-apoptotic genes from CAR-NK cells, thereby increasing their resistance to apoptotic signals within the TME (127). These multifunctional CAR-NK cells, often referred to as “armored” or “NK cell pharmacies,” represent a novel and potentially safer method of locally modulating the TME, which may help minimize or avoid systemic side effects (128).

### 4.4 Integrating CAR-NK therapy with other treatment modalities

Combining CAR-NK cell therapy with conventional treatments like chemotherapy or radiotherapy can produce synergistic effects, enhancing tumor antigen exposure and increasing cancer cells’ susceptibility to immune-mediated destruction. This multifaceted approach not only aims to improve tumor eradication but also reduces recurrence risks and enhances patient outcomes by targeting tumors from different angles (129). Several studies have demonstrated that CAR-T/NK cells maintain their cytotoxic activity in the presence of cisplatin, amplifying their cytotoxic effects while exhibiting potent anti-tumor activity (68, 130). Research has also shown that CD44-targeted CAR-NK cells retain cytotoxicity when combined with cisplatin, with this combination showing greater anti-tumor efficacy than sequential treatments (131). Furthermore, CAR-NK92 cells targeting CD133, when used with cisplatin, exhibit significant anti-tumor effects in ovarian cancer, with cisplatin not affecting the cytotoxicity or viability of CAR-NK cells (68). In addition, the synergistic effects of CAR-T/NK cells combined with radiotherapy have been confirmed in glioblastoma and pancreatic cancer treatments, reinforcing the role of chemotherapy in advancing CAR-NK cell therapy. Radiotherapy remains a key treatment option for many cancers, both curative and palliative. Emerging evidence suggests that radiotherapy, especially stereotactic body radiotherapy, can synergize with immunotherapies like anti-PD-1 or anti-CTLA4 antibodies (132, 133). Preclinical studies suggest mechanisms such as enhanced tumor antigen availability, the release of immunostimulatory cytokines, and disruption of tumor-supportive stroma, all of which aid in recruiting and activating antigen-presenting cells. This can initiate specific tumor immune responses, leading to regression at distant, non-irradiated sites, a phenomenon known as the “abscopal effect” (134). Radiation-induced DNA damage may also cause the expression of NKG2D ligands on tumor cells, potentially boosting NK cell activation and cytotoxicity against these tumor cells (135, 136). Therefore, integrating CAR-NK cell therapy with localized radiotherapy presents a promising strategy, particularly for solid tumors.

Immune checkpoint inhibitors (ICIs) such as anti-PD-1/PD-L1 and anti-CTLA4 antibodies (137) have shown impressive clinical outcomes across various cancers. In particular, PD-1 pathway inhibition has enhanced the efficacy of CAR-T cell therapy (138–140). However, systemic ICI administration may lead to heightened immune-related side effects (141). Recent advancements have focused on delivering anti-PD-1 antibodies or scFv directly to tumor sites using engineered CAR-T cells, enhancing anti-tumor effects while minimizing side effects compared to traditional systemic treatments (142, 143). A recent study on CLDN6-targeted CAR-NK cells found that combining them with anti-PD-L1 antibodies synergistically boosted anti-tumor efficacy, highlighting the potential of pairing CAR-NK cells with PD-1/PD-L1 inhibitors for cancer therapy (80). Additionally, CRISPR/Cas9 technology has been employed to remove the PD-1 gene from CD19-targeted CAR-T cells, improving their efficacy against CD19-positive cancer cells (144). Similarly, knocking down PD-1 in CAR-T cells has reduced T-cell exhaustion and increased the proportion of memory T cells, strengthening anti-tumor immunity (126). These findings emphasize the potential of PD-1 gene editing to enhance CAR-T cell performance, offering a promising approach for designing CAR-NK cells to improve their anti-tumor capabilities. Activated NK cells express various T-cell immune checkpoint molecules like PD-1, CTLA-4, LAG3, and TIM3, which can inhibit their tumor-fighting functions. Inhibiting these checkpoints can substantially enhance NK cell activity (145, 146). Combining CAR-NK cells with systemic or localized ICI therapies or using gene-editing strategies to disrupt checkpoint expression represents an evolving approach for improving CAR-NK cell therapy, particularly in solid tumor treatments.

Moreover, sequential therapy using CAR-NK cells followed by CAR-T cells offers a potent and safe strategy to eliminate tumors in a synergistic and sustained manner, particularly for patients with significant tumor burdens. Readily available “off-the-shelf” CAR-NK cell products can be used immediately to reduce tumor load before administering CAR-T cells, which typically require 2–4 weeks for preparation. Some CAR-T cells can persist as memory T cells, maintaining long-term anti-tumor activity. Consequently, sequential administration of CAR-NK and CAR-T cells could lead to rapid and lasting tumor reduction. Additionally, the initial reduction of tumor burden with CAR-NK therapy may reduce the chances of cytokine release syndrome (CRS) and neurotoxicity during subsequent CAR-T cell therapy, thus minimizing potential adverse effects (147).

## 5 Conclusion and future perspectives

In summary, CAR-NK cell therapy offers great potential as a treatment strategy for solid tumors, capitalizing on the unique properties of NK cells combined with the flexibility of CAR technology. This approach could significantly enhance cancer immunotherapy and improve patient outcomes. However, several challenges persist, including the effective targeting of tumor-specific antigens, overcoming the suppressive effects of the TME, ensuring the long-term persistence of CAR-NK cells, and integrating these cells with other established treatment modalities.

Future efforts should focus on discovering new tumor antigens that can be selectively targeted by CAR-NK cells, thereby improving therapeutic efficacy while minimizing off-target effects. The advancement of genetic tools like CRISPR/Cas9 will enable the creation of more robust CAR-NK cells that can resist immune evasion tactics and thrive in the harsh conditions of the TME. Additionally, exploring combinations of CAR-NK cell therapy with ICIs, targeted therapies, or conventional treatments such as chemotherapy and radiotherapy could significantly enhance overall treatment outcomes. Another key area of research should be improving the expansion and longevity of CAR-NK cells within the body. Strategies to enhance their survival, such as the use of cytokines or genetic modifications, will be essential for maximizing their therapeutic potential in clinical settings. Understanding the dynamics of the TME and how CAR-NK cells can be optimized to navigate these challenges will further strengthen their role in cancer therapy.

In addition to these directions, emerging technologies such as spatial multiomics, single-cell transcriptomics, and high-resolution proteogenomics offer powerful opportunities to further optimize CAR-NK therapy. Spatial transcriptomics and spatial proteomics allow the precise mapping of tumor antigens, stromal components, and immune cell interactions within their native tissue architecture, enabling the identification of spatially restricted or niche-specific targets that may not be captured by bulk sequencing. Moreover, integrating spatial multiomics with single-cell analyses can reveal functional NK cell states, exhaustion trajectories, and cell–cell communication networks within the tumor microenvironment, providing critical insights for engineering CAR-NK cells with enhanced infiltration, persistence, and resistance to immunosuppression. These technologies can also guide the development of next-generation CAR constructs by pinpointing highly specific, spatially conserved tumor epitopes and by uncovering mechanisms of therapeutic resistance. As spatial multiomics continues to advance, it is expected to play an increasingly important role in refining target selection, predicting treatment responsiveness, and informing more precise and personalized CAR-NK cell–based immunotherapeutic strategies.

As CAR-NK cell therapy continues to advance, a multidisciplinary approach that combines immunology, genetic engineering, and oncology will be necessary to translate these innovations into practical treatments. Close collaboration between researchers and clinicians will be vital for realizing the full potential of CAR-NK cell therapy, ultimately paving the way for more effective and personalized cancer treatments.
